# Minor Variant Detection at Different Template Concentrations in HIV-1 Phenotypic and Genotypic Tropism Testing

**DOI:** 10.2174/1874357900802010008

**Published:** 2008-03-18

**Authors:** Ina Vandenbroucke, Veerle Van Eygen, Evelien Rondelez, Hans Vermeiren, Kurt Van Baelen, Lieven J Stuyver

**Affiliations:** Virco BVBA, Generaal De Wittelaan L11 B4, 2800 Mechelen, Belgium

## Abstract

The clinical trials of maraviroc showed that treatment failure was mostly associated with lack of X4 virus detection at baseline. The detection limit for X4 in tropism assays is ill defined around 10%. In the current study, quantification of X4-tropic minority populations was assessed on artificial mixed samples and 38 clinical isolates. These mixtures were subjected to tropism “clonal genotyping” or “population phenotyping”. The detection of minority variants was dependant on the input of amplifiable copies. At VL > 4 log IU/ml, X4 quantification was deemed reliable. PCR founder effect and clonal resampling might result in misrepresentation of the minority species concentration at VL < 4 log. Fourteen of the clinical isolates contained dual/mixed X4-tropic virus, 5 of which were below 10% of the virus population. Currently, there is no indication what level of X4 would lead to treatment failure. Assays aiming for the detection of minority species should express results in function of VL.

## INTRODUCTION

Recently, the FDA has approved maraviroc (Selzentry), the first of a new class of anti-*Human Immunodeficiency virus 1* (HIV-1) therapeutics that targets HIV by blocking its entry into cells [1]. Maraviroc was approved for use in combination with other antiretroviral drugs in HIV-1 experienced adult patients infected with only CCR5-tropic virus. The approval of maraviroc is based on safety and effectiveness data from 24-week data from two double-blind, placebo-controlled studies (MOTIVATE 1 and 2) with over 1000 clinical trial participants [2, 3]. The label indicates that tropism testing and treatment history should guide the use of maraviroc, and that the use of maraviroc is not recommended in patients with dual/mixed or CXCR4-tropic HIV-1 [4].

A phenotypic tropism test (Trofile, Monogram Biosciences, CA; [5]) was used during the MOTIVATE clinical trials to identify patients appropriate for treatment with maraviroc. The sensitivity of the Trofile test was determined after mixing of molecular clones, not viruses, which revealed a 100% sensitivity in picking up 10% X4-variants and 85% sensitivity at the 5% level [5]. A total of 50 to 60% of patients with only R5 viruses were enrolled [2, 3]. Treatment failure on maraviroc was associated with detection of CXCR4-tropic or dual/mixed tropic virus that was not detected by the tropism assay prior to treatment [4, 6]. As the label for maraviroc indicates that prescriptions and use should be guided by tropism testing and treatment history, and for treatment of CCR5-tropic virus only, the presence of X4-tropic virus as detected by assays with cut-offs below 10% brings a new challenge to the treatment and prescription paradigm.

We developed a tropism testing platform including a genotypic and phenotypic procedure at a population and clonal level [7]. The genotypic and phenotypic assays accept amplicons spanning the aminoterminal through the V4-loop (NH_2_-V4) of the HIV-1 Envelope gene. The presence of X4-tropic virus in the quasispecies in several clinical isolates of therapy-naïve individuals was previously illustrated [7].

In the experiments described inhere, we i) further substantiated the presence of X4-tropic virus at low percentages (below 10%) in a random selection of clinical isolates, and ii) analyzed simulations of mixed populations at different viral loads.

## MATERIALS AND METHODOLOGY

### Sample Selection, and Tropism Determination

Properly consented plasma samples (including naïve and therapy-experienced) were randomly selected and analyzed as described in detail in Van Baelen *et al.* [7]. In the genotypic assay: from 29 (out of 38) clinical isolates a total of 95 individual clones were analyzed, and an average of 80 ± 7 (range 62-88, Table **2**) clones gave quality approved sequences. For the remaining 9 (out of 38) samples, 47 recombinant clones were analyzed, resulting in 40 ± 7 (range 23 – 47, Table **2**) sequences useful for tropism prediction using PSSM

(http://ubik.microbiol.washington.edu/computing/pssm/) and SVM (http://co-receptor.bioinf.mpi-sb.mpg.de/cgi-bin/co-receptor.pl).

### Preparation of Plasmids and Recombinant Virus Stocks (RVS)

The gp120 region from pNL4.3 (X4-tropic; Genbank Acc. No. AF324493) or pYK-JRCSF (R5-tropic; Genbank Acc. No. M38429) was amplified and cloned into pHXB2D-ΔNH_2_-V4-eGFP as described [7]. Recombinant virus stocks (RVS) were generated by plasmid nucleofection (Amaxa Biosystems, Cologne, Germany) as described [7] except that precautions were taken to remove contaminating plasmid DNA by washing the cells 16 hours post nucleofection. The RVS (referred to as recNL4.3 and recJRCSF) were harvested 48 hours post-infection.

### Quantification of Viral RNA in RVS

Viral load (VL) of recJRCSF and recNL4.3 RVS was determined on a 1/10 dilution series using the EasyMAG - Nuclisens EasyQ HIV-1 method (Biomérieux, Boxtel, The Netherlands). Viral load values obtained were averaged and revealed that the recJRCSF RVS and recNL4.3 RVS contained 8.7 +/- 0.16 log IU/ml and 9.4 +/- 0.13 log IU/ml, respectively.

### Quantification of Plasmid DNA in RVS

The presence of plasmid DNA, relative to the total nucleic acid content, was measured using real-time (RT-)PCR. RNA was quantified using primer HIV-1-F (5’-TGGGTTAT GAACTCCATCCTGAT-3’), HIV-1-R2 (5’-TGTCATTGA CAGTCCAGCTGT-3’) and probe (6-FAM-TTTCTGGCA GCACTATAGGCTGTACTGTCCATT-TAMRA). Similarly, DNA was quantified in experiments without reverse transcriptase added. recJRCSF and recNL4.3 RVS RNA isolates contained 0.31 % and 0.39 % copies plasmid, respectively.

### Preparation of Mixtures of RVS, and RNA Extraction

Appropriate amounts of quantified recJRCSF and recNL4.3 RVS were used to reconstitute a set of viral mixes to yield final recNL4.3 fractions of 2.5% and 1% at 5 log IU/ml, 20% and 8% at 4 log IU/ml, and 20% at 3 log IU/ml. RNA extraction was performed on 3 to 6 aliquots of 300 µl each (EasyMAG).

### Restriction Analysis

*Alu*I restriction digestion was performed on the colony PCR products in order to distinguish between clones from recNL4.3 (4 fragments) or recJRCSF (3 fragments).

## RESULTS

### Tropism Analysis of Reconstituted Samples According to Viral Load

Since the exact composition of the viral quasispecies in clinical isolates is unknown, we aimed to mimic minority species conditions on artificial lab-generated virus mixtures.

**Fig. (1):**
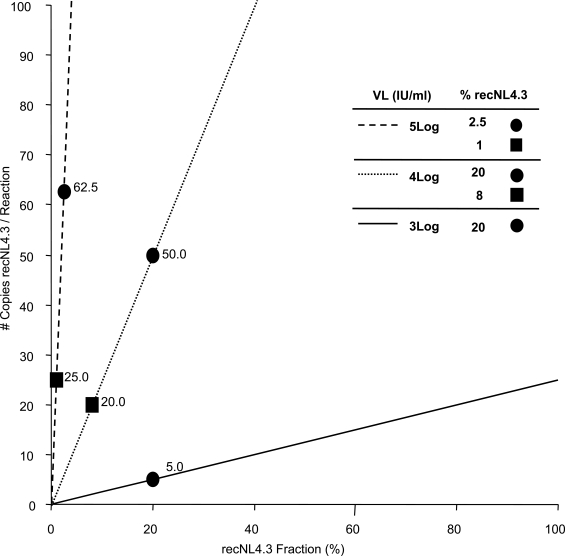
Visualization of the theoretical number of copies of recNL4.3 in each RT-PCR reaction. X-axis: percentage of the minority species recNL4.3 in the total amount of amplifiable RNA genomes; Y-axis: number of copies of the minority species per RT-PCR reaction. The number of copies per reaction is function of the viral load and experimental procedure. Dashed line: 5 log VL; dotted line: 4 log VL; solid line: 3 log VL. Experimental conditions (viral load, sample amount in RNA extraction, RNA input in RT-PCR) influence the slope of the lines. This figure represents the applied conditions.

The theoretical number of recNL4.3 copies, present in each RT-PCR reaction was calculated for different viral loads, and plotted as a function of the fraction recNL4.3 in the original viral mixture (Fig. **1**). From this Figure one can deduce that, if, for example, the extraction/ amplification procedure starts with 300 μl plasma, eluted in 60 μl H_2_O, of which 5 μl is used in RT-PCR, the 20% minority fraction at 3 log IU/ml corresponds to only 5 copies. Hence, the ability to detect minority quasispecies depends on initial viral load and assay design (amount of RNA copies used for RT-PCR).

Subsequently, artificial viral mixtures panels were analyzed as visualized in Fig. (**2**), and results are shown in Table **1**. At 5 log IU/ml, a 1% minority variant was detected in one out of three repeat experiments, while a 2.5% minority variant was detected in four out of six repeat experiments. At 4 log IU/ml, the minority detection success rate was 6 out of 6 (100%) for an 8% and 20% minority population. At 3 log IU/ml, the 20% minority variant was detected in three out of five experiments. At 5 and 4 log IU/ml, fluctuations between the different outcomes of the parallel experiments were relatively low, while at 3 log IU/ml, detected quantities were very different and varied from 0 to 47.3%. This can be ascribed to the initial founder effect in PCR reactions with low copy numbers, and visualized/enhanced by resampling during colony picking. When analyzing *n* random amplification products from an RT-PCR performed with a sample containing *D* RNA molecules, the expected number of independent clones (*E*) is given by **E = D(1-((D-1)/D)^n^)** [8, 9]. For example at 3 log IU/ml, 175 RNA copies (= D) were inserted into the RT-PCR and 93 clones were picked (= n), hence E = 175(1-((175-1)/175)^93^) = 72. Applied to our experimental conditions, resampling might occur at a viral load of approximately 4 log or less. A selection of the samples was also tested in the population-based phenotypic analysis. At 5 log IU/ml, a 1% X4-tropic minority species was detected (Table **1**). In the 2.5% recNL4.3 experiment in which no recNL4.3 could be detected genotypically, there was also no X4-tropic virus detected by phenotypically. At 4 log IU/ml, the minority species were detected in all experiments; at 3 logIU/ml, the minority was detected in two out of three experiments.

From these experiments the following conclusions were made: i) qualitative detection of the minority species of the total population is possible genotypically and phenotypically between 3 to 5 log IU/ml; ii) quantification of the minority species is possible genotypically, but only if the viral load is higher than 4 log IU/ml, and it requires multiple separate amplifications and a high amount of colonies to analyze (Table **1**); iii) quantification of the minority species in samples with a viral load below 4 log IU/ml is technically challenging and it might contain the bias of founder effect amplification (PCR drift).

### Tropism Analysis of Clinical Samples

With the availability of genotyping and phenotyping protocols – including a minority species quantification if viral load is > 4 log of IU/ml in the genotypic assay - a total of 38 random clinical samples were analyzed (Table **2**). In 39% of the samples (15/38), both algorithms predicted the presence of X4, of which 7 (47%) had X4 frequencies below 10%, and 4 samples (27%) below 5%. Five out of the 7 samples with X4 virus below 10% (samples 25, 26, 27, 32 and 33) were resulting in the population–based phenotyping as dual/mixed tropic (D/M) virus. Two samples that contained a subpopulation of X4 predicted clones at ± 2.5% (samples 1 and 2) resulted in the phenotypic assay as R5-tropic only. From another 19 samples, clonal sequences with discordant predictions were obtained, but all of these belonged to samples that were phenotypically R5-only. One sample contained D/M virus (sample 37), which could not be detected using genotypic analysis.

The formula E = D(1-((D-1)/D)^n^) [8, 9] was also applied to these experiments (Table **2**), again illustrating that resampling occurred at a viral load of approximately 4 log or less. Especially in the D/M samples with low copy number input, PCR founder effects and resampling could bias the results and might lead to high percentages of X4 clonal sequences. Also, the detection of the minor percentages of X4 tropic virus was only in samples with a viral load higher than 4 log IU/ml.

The conclusions of these experiments were as follows: i) X4 tropic virus is often present in clinical isolates at low percentages (<10% of the total population) and might remain undetected in some routine diagnostic tests; ii) below 4 log IU/ml, genotypic results are qualitative; iii) the qualitative phenotypic assay applied in these experiments detects most of the X4 predicted genotypes; and iv) several hundreds of genotypic clones were found discordant in the prediction algorithms (as was also recently described [10]).

## DISCUSSION

CCR5 antagonists belong to the co-receptor antagonist drug class. Maraviroc, also known as Selzentry or Celsentri, is the first to receive accelerated approval by the FDA for use in combination therapy in adults infected with only CCR5-tropic HIV-1. Clinical resistance to maraviroc has not yet been fully defined, but virologic failure has been associated with the unmasking of X4-tropic virus by reducing the R5-tropic majority population, currently also interpreted as viral tropism switches [4]. The FDA states that screening for X4 viruses by HIV-1 tropism testing prior to therapy with a coreceptor antagonist should guide the use of maraviroc [4]. Several assays are available (commercially and non-commercially) that determine coreceptor usage. Some of these are based on genotypic analysis of (parts of) the envelope gene, which are either sequenced and interpreted with an algorithm [7, 11], or tropism detection is based on differential hybridization of an amplicon to a probe set [12, 13]. Genotypic assays are generally fast and cheap. They rely however on the current knowledge about mutations and polymorphisms associated with co-receptor usage (interpretation algorithms or reference DNA probes). As the correlative databases will increase and algorithms will be improved, these methods might gain in importance. Phenotypic methods are based on viral entry into host cells either containing the CCR5 or CXCR4 co-receptor. Recombinant viruses are prepared by cloning or recombining (part of) the envelope gene into an HIV-1 backbone deleted for the corresponding amplicon. They consist either of a single-cycle assay (Trofile and Phenoscript [5, 14, 15] or multiple cycle assay (PhenXR and Antivirogram [7, 12]). The limit of detection for X4-virus in the single cycle assays is around 5-10% [5, 16]. It is not clear what level of X4-tropic virus would lead to treatment failure and what degree of X4 increase is reason for concern.

Sensitivity of an assay towards detection of a minor population is assessed in some studies by analyzing mixtures of amplicons [17] or plasmids [5, 18]. Extraction and amplification of viral RNA was not included in such evaluations, although these steps are an integral part of the assay workflow and contribute crucially and significantly to its sensitivity. We mimicked clinical isolates by mixing an X4-tropic minority variant with R5 tropic virus at different viral loads. The proportion of minority variants that can be detected is inversely proportional to the viral load of the sample, as theoretically anticipated [19]. It might be unrealistic to expect detection of minority variants below 1% at 5 log IU/ml, with decreasing chance of success at even lower viral load.

At 3 log IU/ml, and a 20% minority component, expected *vs* measured outcomes differed severely. This bias in template-to-product ratio is known as PCR drift and is caused by stochastic variation in the early cycles of the reaction, leading to a deviating estimation of the template frequency, especially at low copy numbers [20, 21]. In addition, the calculated number of copies present in the sample might be substantially lower than measured by the VL assay, which is based on amplification of a very small fragment (± 80 bp). In contrast, the amplification of the longer NH_2_-V4 fragment (± 1,3 Kb) is much more dependent on the presence of intact RNA.

A second problem at low viral load might be the issue of resampling [9]. Resampling occurs if one or only a few molecules of the sample are used as input for the PCR and results in repeated analysis of PCR products amplified from the same amplifiable viral RNA molecule. This can result in artificially low levels of viral diversity, not representative for the original sample. To avoid resampling, the maximal amount of analyzed clones should not exceed the number of inserted copies into the RT-PCR [8, 9].

There is growing evidence that minor X4 subpopulations are common among HIV-1 patients [22, 23]. As viral tropism determination assays will become more mature and more sensitive, X4-tropic virus will be detected in many more patients samples. It needs to be determined what the clinical significance of these populations is and what the implications are for co-receptor antagonist use. Our results show the quantification limitations in PCR technologies on samples with a low amount of amplifiable genomes. This phenomenon is inherent to all PCR-based assays (including testing for protease and RT gene resistance mutations) on small amounts of complex templates [24] and should be carefully considered when samples are analyzed with low input copy number. Assays aiming for minority species detection are limited by the amount of copies inserted into the RT-PCR procedure and should express results in function of the number of target molecules present in the original sample.

## Figures and Tables

**Fig. (2):**
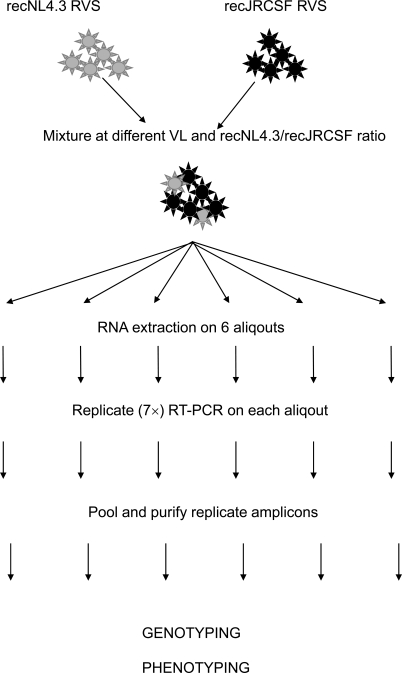
Workflow for genotypic and phenotypic analysis of the recJRCSF/recNL4.3 mixtures. Two RVS were mixed at different ratios and viral loads. Each mixture was split into 6 aliquots and processed in parallel. RNA was extracted of each aliquot, followed by 7 replicate amplifications of the NH_2_-V4 region. PCR products were pooled, purified and cloned. Colonies were picked, the insert was PCR amplified, purified and analyzed by *Alu*I restriction analysis. Tropism was assessed by population-based phenotyping as described [7]. Infection of CCR5- and CXCR4-expressing U87-cells was scored using a fluorescent microscope and FACS.

**Table 1.** Overview of Genotypic and Phenotypic Testing of Artificial Mixed Samples at Various Viral Loads

Sample Characteristics			Clonal Genotype^b^					E^d^	Phenotype^e^
VL (log IU/ml)	# cp in 7x RT-PCR^a^	X4 present (%)	# Clones Analyzed	R5	X4	Discordant^c^	X4 Measured (%)		
5	17500	2.5	93	91	2	0	2.2	93	
5	17500	2.5	92	90	2	0	2.2	92	
5	17500	2.5	93	93	0	0	0.0	93	
5	17500	2.5	94	94	0	0	0.0	94	R5
5	17500	2.5	95	91	4	0	4.2	95	D/M
5	17500	2.5	93	91	2	0	2.2	93	D/M
5	17500	1	95	95	0	0	0.0	95	
5	17500	1	94	94	0	0	0.0	94	
5	17500	1	94	93	1	0	1.1	94	D/M
4	1750	20	92	82	10	0	10.9	***90***	
4	1750	20	93	88	5	0	5.4	***91***	
4	1750	20	92	85	7	0	7.6	***90***	
4	1750	20	95	87	8	0	8.4	***92***	D/M
4	1750	20	91	78	13	0	14.3	***89***	D/M
4	1750	20	94	92	2	0	2.1	***92***	D/M
4	1750	8	94	86	8	0	8.5	***92***	
4	1750	8	95	92	3	0	3.2	***92***	
4	1750	8	92	85	7	0	7.6	***90***	
4	1750	8	94	92	2	0	2.1	***92***	
4	1750	8	92	89	3	0	3.3	***90***	D/M
4	1750	8	92	84	8	0	8.7	***90***	D/M
3	175	20	95	95	0	0	0.0	***73***	
3	175	20	89	89	0	0	0.0	***70***	
3	175	20	90	86	4	0	4.4	***71***	R5
3	175	20	94	93	1	0	1.1	***73***	D/M
3	175	20	93	49	44	0	47.3	***72***	D/M

a Number of viral copies entered in procedure.

b Tropism was inferred from the generated restriction pattern.

c Number of discordant clones between PSSM and SVM; all clones were predicted to be R5-tropic in PSSM, but either dual-tropic, X4-tropic or undetermined in SVM.

d E value obtained from the formula E = D(1-((D-1)/D)^n^) representing the expected number of independent clones, *n* = number of analyzed clones; *D* = number of input RNA copies. Numbers in bold italics indicate conditions where the amount of clones analyzed was greater than the expected number of individual amplifiable genomes present, suggesting resampling conditions.

e Population based phenotypic results obtained on same amplicon used for clonal analysis; D/M: dual tropic or mixed tropic virus population.

**Table 2.** Overview of Genotypic and Phenotypic Testing of 38 HIV-1 Infected Patient Samples at Various Viral Loads

Sample Characteristics			Clonal Genotype^b^					E^d^	Phenotype^e^
Sample ID	VL (log IU/ml)	# cp in 7x RT-PCR^a^	# Clones Analyzed	R5	X4	Discordant^c^	X4 Measured (%)		
1	5.86	126,776	84	0	2	82	2.4	84	R5
2	5.36	40,250	76	0	2	74	2.6	76	R5
3	6.58	665,331	86	0	0	86	0.0	86	R5
4	6.04	192,500	81	54	0	27	0.0	81	R5
5	5.53	59,500	87	86	0	1	0.0	87	R5
6	5.26	31,500	78	78	0	0	0.0	78	R5
7	5.03	18,614	62	11	0	51	0.0	62	R5
8	5.02	18,200	38	37	0	1	0.0	38	R5
9	4.93	14,893	41	11	0	30	0.0	41	R5
10	4.59	6,825	43	20	0	23	0.0	43	R5
11	4.57	6,550	45	45	0	0	0.0	45	R5
12	4.34	3,798	40	38	0	2	0.0	40	R5
13	4.13	2,341	82	81	0	1	0.0	***81***	R5
14	3.97	1,624	84	3	0	81	0.0	***82***	R5
15	3.90	1,379	38	38	0	0	0.0	***37***	R5
16	3.86	1,279	86	0	0	86	0.0	***83***	R5
17	3.83	1,183	87	0	0	87	0.0	***84***	R5
18	3.81	1,129	84	0	0	84	0.0	***81***	R5
19	3.31	361	88	0	0	88	0.0	***78***	R5
20	3.29	343	44	0	0	44	0.0	***41***	R5
21	2.71	90	62	0	0	62	0.0	***45***	R5
22	2.48	53	81	79	0	2	0.0	***42***	R5
23	2.47	52	47	0	0	47	0.0	***31***	R5
24	2.14	24	87	0	0	87	0.0	***24***	R5
25	6.23	297,500	71	2	7	62	9.9	71	D/M
26	6.15	245,000	71	0	5	66	7.0	71	D/M
27	6.11	227,500	76	1	7	68	9.2	76	D/M
28	5.90	140,000	76	0	10	66	13.2	76	D/M
29	5.83	118,315	83	0	12	71	14.5	83	D/M
30	5.82	115,621	85	0	23	62	27.1	85	D/M
31	5.41	44,982	86	0	11	75	12.8	86	D/M
32	5.20	28,000	76	0	3	73	3.9	76	D/M
33	5.18	26,250	84	0	1	83	1.2	84	D/M
34	4.91	14,225	86	0	10	76	11.6	86	D/M
35	4.43	4,710	76	12	64	0	84.2	***75***	D/M
36	2.92	146	87	30	57	0	65.5	***66***	D/M
37	2.66	79	23	20	0	3	0.0	***20***	D/M
38	2.19	27	86	0	84	2	97.7	***26***	D/M

a Number of viral copies entered in procedure.

b Tropism determination by clonal genotyping. Prediction was performed on the clonal V3 loop sequences using the PSSM and SVM on-line available software

c Number of discordant clones between PSSM and SVM; all clones were predicted to be R5-tropic in PSSM, but either dual-tropic, X4-tropic or undetermined in SVM.

d E value obtained from the formula E = D(1-((D-1)/D)^n^) representing the expected number of independent clones, *n* = number of analyzed clones; *D* = number of input RNA copies. Numbers in bold italics indicate conditions where the amount of clones analyzed was greater than the expected number of individual amplifiable genomes present, suggesting resampling conditions.

e Population based phenotypic results obtained on same amplicon used for clonal analysis; D/M: dual tropic or mixed tropic virus population.
